# Early Pharmacodynamic Changes Measured Using RNA Sequencing of Peripheral Blood from Patients in a Phase I Study with Mitazalimab, a Potent CD40 Agonistic Monoclonal Antibody

**DOI:** 10.3390/cells12192365

**Published:** 2023-09-27

**Authors:** Hampus Andersson, Aastha Sobti, David Gomez Jimenez, Yago Pico de Coaña, Sumeet Vijay Ambarkhane, Karin Hägerbrand, Karin Enell Smith, Malin Lindstedt, Peter Ellmark

**Affiliations:** 1 Alligator Bioscience AB, 223 81 Lund, Sweden; haa@alligatorbioscience.com (H.A.); aso@alligatorbioscience.com (A.S.); daj@alligatorbioscience.com (D.G.J.); yas@alligatorbioscience.com (Y.P.d.C.); malin.lindstedt@immun.lth.se (M.L.); 2 Department of Immunotechnology, Lund University, 223 81 Lund, Sweden

**Keywords:** CD40, mitazalimab, pharmacodynamics, RNA sequencing, cancer

## Abstract

CD40-targeting therapies can enhance the dendritic cell priming of tumor-specific T cells and repolarize intratumoral macrophages to alleviate the tumoral immunosuppressive environment and remodel the extracellular matrix. Mitazalimab is a potent agonistic CD40 monoclonal IgG1 antibody currently under clinical development. This study used RNA sequencing of blood samples from a subset of patients from a Phase I trial with mitazalimab (NCT02829099) to assess peripheral pharmacodynamic activity. We found that mitazalimab induced transient peripheral transcriptomic alterations (at 600 µg/kg and 900 µg/kg dose administered intravenously), which mainly were attributed to immune activation. In particular, the transcriptomic alterations showed a reduction in effector cells (e.g., CD8^+^ T cells and natural killer cells) and B cells peripherally with the remaining cells (e.g., dendritic cells, monocytes, B cells, and natural killer cells) showing transcription profiles consistent with activation. Lastly, distinct patient subgroups based on the pattern of transcriptomic alterations could be identified. In summary, the data presented herein reinforce the anticipated mode of action of mitazalimab and support its ongoing clinical development.

## 1. Introduction

The advent of immune checkpoint inhibitors (ICIs) has profoundly transformed cancer therapy, offering a potential remedy for individuals with advanced or metastatic cancer [1]. However, the efficacy of ICI therapy relies on the presence of an existing immune response within the tumor microenvironment (TME), including the infiltration of CD8^+^ T cells. Consequently, tumors that lack sufficient T-cell infiltration or exhibit impaired T-cell priming remain a significant unmet clinical need [2]. Furthermore, many tumors, e.g., pancreatic tumors, contain a desmoplastic stroma that hosts suppressive myeloid cells such as tumor-associated macrophages that dampen the immune response in the TME [3]. The ability to overcome these challenges may significantly improve the efficacy and outcome of existing and future treatment principles in solid tumors.

CD40, a member of the tumor necrosis factor receptor superfamily, is expressed on the surface of immune cells (e.g., dendritic cells (DCs), B cells, and macrophages) as well as on other cell types such as epithelial, endothelial, and neoplastic cells [4]. Signaling through CD40 promotes the ability of DCs to prime T-cell responses [5], and therapies targeting CD40 have emerged as a promising strategy to overcome resistance to ICIs [6,7]. CD40-targeting therapies aimed at augmenting the priming of tumor-specific T cells have the potential to increase response rates in approved indications where ICI therapies have limited efficacy, as well as expand the scope of eligible immunotherapy indications [1], by making the tumors more inflamed via pathways such as type I interferon [8], effectively turning immunological cold tumors hot [9]. Additionally, CD40 signaling in macrophages can potentially alleviate the immunosuppressive TME and remodel the extracellular matrix [6,10]. Clinical evidence supporting the benefits of immunotherapy with agonistic CD40 antibodies, either as monotherapy or in combination therapy, is emerging, and second-generation CD40 agonists are currently under clinical development [6,7,11,12].

Mitazalimab, a CD40-targeting agonistic human monoclonal IgG1 antibody (mAb), is being developed as immunotherapy for advanced solid tumors. Preclinically, mitazalimab has shown significant antitumor activity and long-term tumor-specific immunity in human CD40-transgenic mice as well as antitumor activity in immune-deficient NSG mice [13]. Clinically, mitazalimab has been proven to have a manageable safety profile and pharmacodynamic activity consistent with its proposed mechanism of action and demonstrated promising clinical activity in an ongoing Phase 2 study in metastatic pancreatic cancer (OPTIMIZE-1, NCT04888312) [14,15]. Pharmacodynamic biomarkers related to mitazalimab have been characterized both preclinically and clinically and include transient reduction in circulating B cells, T cells, NK cells, and monocytes, where the remaining B cells are more activated, consistent with other CD40 agonists [13,14,15,16,17,18].

In this study, the peripheral transcriptome of a subset of patients from a Phase I clinical study of mitazalimab (NCT02829099) was explored for pharmacodynamic activity. We found that mitazalimab transiently induces peripheral transcriptomic changes attributed to immune activation, reinforcing its mode of action as a CD40 agonist. Furthermore, the transcriptomic data enabled patient stratification, unveiling distinct alterations induced by mitazalimab in two different patient groups.

## 2. Materials and Methods

### 2.1. Study Population and Design

A subset of 38 patients from a Phase I, multicenter, open-label, dose-escalation study of mitazalimab in patients with failed standard treatment for confirmed advanced or refractory solid malignancy (ClinicalTrials.gov: NCT02829099; Eudra CT: 2016–000969-23), was selected for analysis using RNA sequencing. The study design and the results of the clinical trial have previously been reported [15]. The subset of patients included in this study received mitazalimab intravenously at four different doses (75, 200, 600, or 900 µg/kg) every two weeks in 28-day treatment cycles. Moreover, a subpopulation (*n* = 5) of the included patients at 600 µg/kg received pretreatment with corticosteroids, as described previously [15].

### 2.2. RNA Sequencing of Blood Samples

For bulk mRNA sequencing, blood samples were collected at baseline (cycle 1 day 1, C1D1), 24 h after the first mitazalimab administration (cycle 1 day 2, C1D2), and before starting the third treatment cycle (cycle 3 day 1, C3D1). RNA was extracted, processed to reduce globin RNA, and prepared for sequencing using an Illumina TruSeq Stranded mRNA sample preparation kit. Samples were sequenced using a 2 × 50 base pair paired-end sequencing protocol, aligned to references genome using STAR (v2.4), and quantified using RSEM (v1.2.14). The data were further analyzed in R (v4.2.2) and R Studio (v 2023.06.0+421). R scripts and data to reproduce the data analysis are available in a repository on GitHub upon request.

Gene counts matrix was analyzed to remove low expressing genes and for samples with large inter-individual Z-score distribution. The 1500 most variable genes based on standardized variance were selected for principal component analysis (PCA). The effects of confounding variables were assessed by calculating the correlation between principal components (PCs) and metadata variables from the study, using the DEGreport package (v1.34) [19]. Raw gene counts were used for differential gene expression (DGE) analysis using DESeq2 (v1.38.3) [20] correcting for repeated inter-patient sampling. Genes with absolute log_2_ fold changes of >1 and *p*-value of <0.05 were, if not otherwise stated, considered differentially expressed. The visualization of differentially expressed genes (DEGs) was carried out with the EnhancedVolcano package (v1.16) [21].

Heatmaps of gene expression were generated from the Z-scores of VST gene counts using ComplexHeatmap (v2.14) [22]. If not otherwise stated, clustering related to heatmaps was performed using the complete agglomeration of Euclidean distances. For k-mean clustering on heatmaps, the optimal number of clusters was calculated using the Elbow method to minimize the sum of squares within clusters [23]. Gene set enrichment analysis (GSEA) was performed using enrichR (v3.1) [24] by querying the Gene Ontology database (GO, GO_Biological_Processes_2018). Gene set variation analysis (GSVA) was performed on enriched GO, to quantify the differences between conditions, using the GSVA package [25]. For the deconvolution of cell types, gene counts were transformed to transcripts per million (TPM) and analyzed using CIBERSORTx [26] from the IOBR package (v0.99.9) [27]. The hierarchical clustering of samples was performed using the pvclust package to provide *p*-values using multiscale bootstrap resampling (v2.2) [28]. Correlation to mouse expression was performed using Pearson correlation, as described in Supplementary Materials.

### 2.3. Cytokine Analysis

Blood samples were collected at baseline and 1, 4, and 24 h after mitazalimab treatment. Samples were analyzed for serum chemokines and cytokines (interferon gamma-induced protein (IP)-10, monocyte chemotactic protein (MCP)-1, macrophage inflammatory protein (MIP)-1α, MIP-1β, and interleukin (IL)-10), as previously described [15]. The data were subsequently analyzed and visualized using R and R Studio.

### 2.4. Statistical Testing

If not otherwise stated, the statistical analysis of parametric numerical data with two groups was performed using Student’s *t*-test, and non-parametric numerical data with three or more groups was performed using the Kruskal–Wallis test with Dunn’s test as post hoc analysis. Bonferroni correction was applied as multiple comparison correction. Categorical data were analyzed using the Chi-square test. *p*-values ≤ 0.05 were deemed significant.

## 3. Results

### 3.1. Mitazalimab Induces Transient Peripheral Transcriptomic Alterations in Patients with Advanced Solid Tumors

We analyzed RNA sequencing data from a subgroup of patients from four selected dose levels, with a total of 38 patients, from a clinical Phase I study investigating intravenous mitazalimab administration at escalating doses. The patients had a median age of 56 years (range 18–77 years) and had undergone a median of three previous systemic treatments (range 0–9) for their malignancies and had exhausted their standard treatment options prior to participating in the study with mitazalimab (Table 1). Indications were categorized into 17 different solid malignancies, with sarcoma and breast cancer being the most common indications (13% and 11% of patients, respectively).

**Figure 1:**
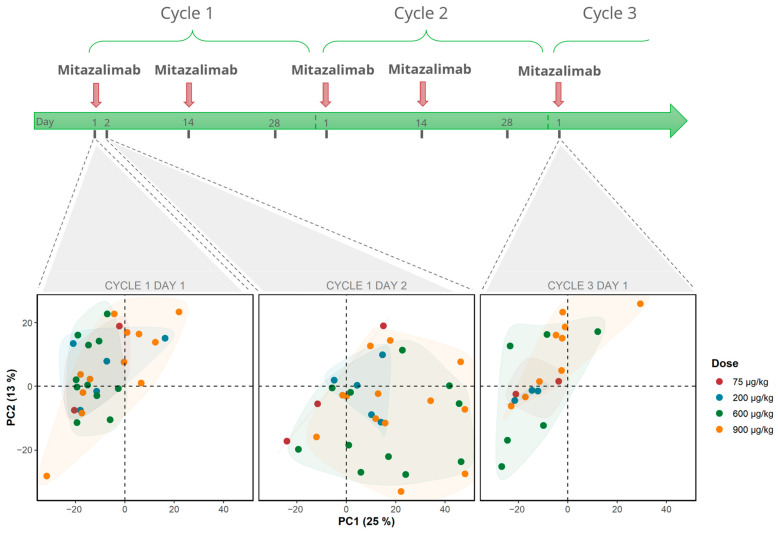
Schematic overview of study design and principal component analysis (PCA) of all samples, not pretreated with corticosteroids, at all analyzed time points. Mitazalimab induced a transient shift along both principal components (PC1 and PC2) which was most predominant at 600 µg/kg and 900 µg/kg. Colors indicate mitazalimab dose, and PC1 and PC2 explain 25% and 13% variance, respectively.

To assess the effects of mitazalimab, we first analyzed patients who did not receive pretreatment with corticosteroids (*n* = 33). Initially, blood samples collected from all four mitazalimab doses at baseline, 24 h post-mitazalimab treatment, and prior to starting the third cycle were analyzed using PCA (Figure 1). At baseline, all samples clustered close to the top-left quadrant of PC1 and PC2 (explaining 25% and 13% of the variation, respectively), thus indicating global transcriptomic similarity at baseline. Following mitazalimab treatment at 600 µg/kg and 900 µg/kg, a notable shift along both PC1 and PC2 towards the bottom-right quadrant was seen, suggesting mitazalimab-induced transcriptomic changes. However, patients treated with 75 µg/kg or 200 µg/kg did not show any obvious changes in PC1 or PC2, indicating a suboptimal pharmacodynamic activity of mitazalimab at these dose levels. In support of these observations, differential gene expression analysis between C1D1 and C1D2 in 75 µg/kg and 200 µg/kg cohorts showed few DEGs (Figure S1). Lastly, prior to starting the third treatment cycle, the samples clustered toward the upper-left quadrant of PC1 and PC2 in an equivalent manner as at baseline, suggesting that the peripheral transcriptomic alterations induced by mitazalimab were transient.

### 3.2. Immune Activation Induced by Mitazalimab Is Detectable in Blood Transcriptome

Next, the transcriptomic changes induced by mitazalimab treatment were investigated. To this end, the patient cohorts treated with 600 µg/kg or 900 µg/kg mitazalimab were further studied, as no evident changes were seen at 75 µg/kg or 200 µg/kg. In DGE analysis between C1D1 and C1D2, the 600 µg/kg and 900 µg/kg cohorts had 1027 and 485 DEGs, respectively. However, no DEGs were found between the 600 µg/kg and 900 µg/kg cohorts at C1D2 (Figure S1), thus suggesting that the difference in the number of DEGs between the groups is attributed to differences in patient characteristics and that the two doses of mitazalimab induce a similar acute effect on the peripheral transcriptome.

**Figure 2:**
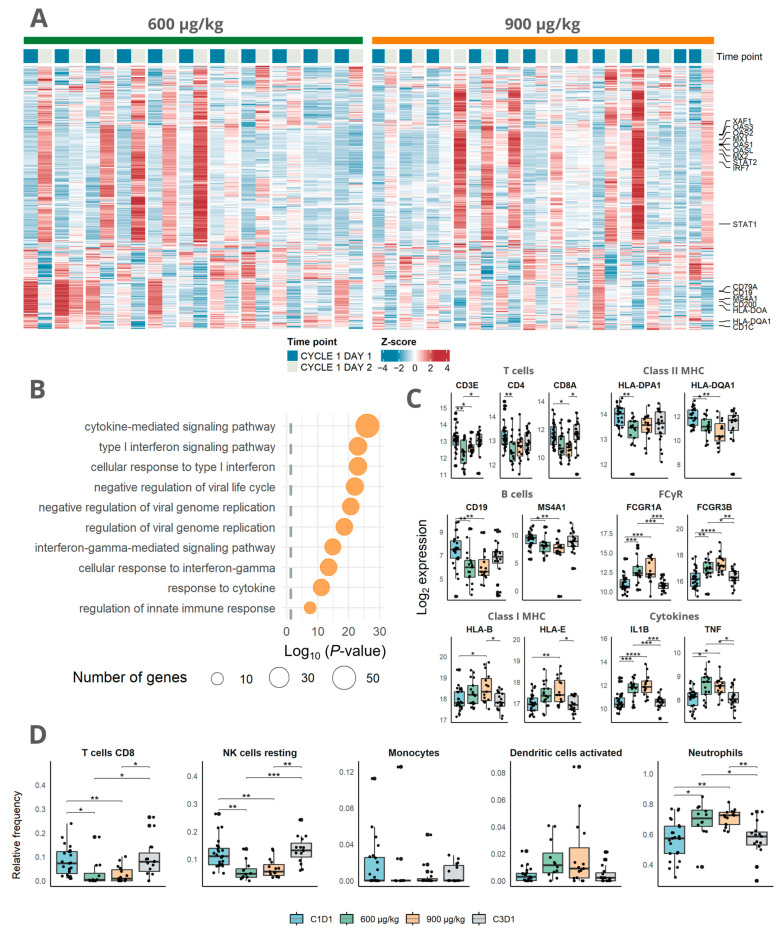
Pharmacodynamic effects on peripheral transcriptome induced by mitazalimab at 600 µg/kg and 900 µg/kg: (**A**) Heatmap of Z-score normalized VST transformed gene expression of the 392 DEGs induced at both 600 µg/kg and 900 µg/kg. Paired columns show paired samples during pretreatment and 24 h post-treatment. (**B**) Pathway enrichment analysis of the 392 shared differentially expressed genes in (**A**). The X-axis shows the log_10_ *p*-value and the dot size indicates the number of genes represented in each pathway. (**C**) Box plots of selected genes illustrate the effect of mitazalimab on immune cells, particularly on effector cells and antigen-presenting cells. Dots represent the expression values of individual patients. (**D**) Relative frequencies of deconvoluted gene types as estimated using CIBERSORTx at C1D1, for each dose cohort at C1D2, and at C3D1. Dots represent individual values. * *p* < 0.05; ** *p* < 0.01; *** *p* < 0.001; **** *p* < 0.0001.

Of the DEGs in the two dose cohorts, 392 DEGs were in common for both dosing cohorts (Figure 2A). Among the most highly upregulated genes in both dosing cohorts were the genes typical of immune activation, such as *CD274* (PD-L1) and *LAMP3* (Table S1). Pathway enrichment analysis for the gene ontology of the commonly DEG genes revealed 378 differentially regulated pathways of which 319 were related to upregulated genes (Table S1). The most significantly differentially regulated pathways among the upregulated genes were the cytokine-mediated signaling pathway (GO:0019221) and type I interferon signaling pathway (GO:0060337; Figure 2B). Together, this indicates that immune activation consistent with the mode of action of mitazalimab could be detected using RNA sequencing in peripheral blood.

To further evaluate the biological effects of mitazalimab, we investigated a set of well-defined immune transcripts. After treatment with either 600 µg/kg or 900 µg/kg, it was found that the genes related to T cells (*CD3E*, *CD4*, and *CD8A*), MHC class II (*HLA-DQA1* and *HLA-DPA1*), and B cells (*CD19* and *MS4A1*) were expressed at a lower level, while the genes related to Fcγ receptors (*FCGR1A* and *FCGR3B*), MHC class I (*HLA-B* and *HLA-E*), and pro-inflammatory cytokines (e.g., *IL1B* and *TNF*) were more highly expressed than at baseline (Figure 2C). Additionally, CIBERSORTx was used to deconvolute gene expression to cell-type abundances. This revealed a relative decrease in signature scores of monocytes, CD8^+^ T cells, and NK cells and an increase in neutrophils and activated dendritic cells in blood following mitazalimab treatment at 600 µg/kg and 900 µg/kg (Figure 2D), suggesting the activation and migration of immune cells in line with the mode of action. In addition, peripheral transcriptomic changes upon mitazalimab treatment in patients correlate well with transcriptomic changes in the preclinical in vivo model (Figure S2).

The potential influence of pretreatment with corticosteroids on the effects of mitazalimab was investigated in patients receiving 600 µg/kg mitazalimab with (*n* = 5) or without (*n* = 15) corticosteroids. PCA revealed a less pronounced mitazalimab effect along PC1 in patients with corticosteroid pretreatment and with a tendency of separation between patient cohorts along PC4 (8% variability; Figure S3A). Differential gene expression analysis showed higher expression of genes typically related to immune activation, such as *LAMP3*, in patients who had not been pretreated with corticosteroids (Figure S3B and Table S2). This was further supported by pathway enrichment analysis indicating that the most significantly differentially regulated pathways among genes with higher expression in patients without corticosteroid pretreatment were the cytokine-mediated signaling pathway (GO:0019221) and type I interferon signaling pathway (GO:0060337; Figure S3C). Overall, the results show that mitazalimab treatment without corticosteroid pretreatment induced stronger inflammatory gene expression.

### 3.3. Transcriptomic Analysis Revealed a Distinct Response Pattern in a Subset of the Patients

**Figure 3:**
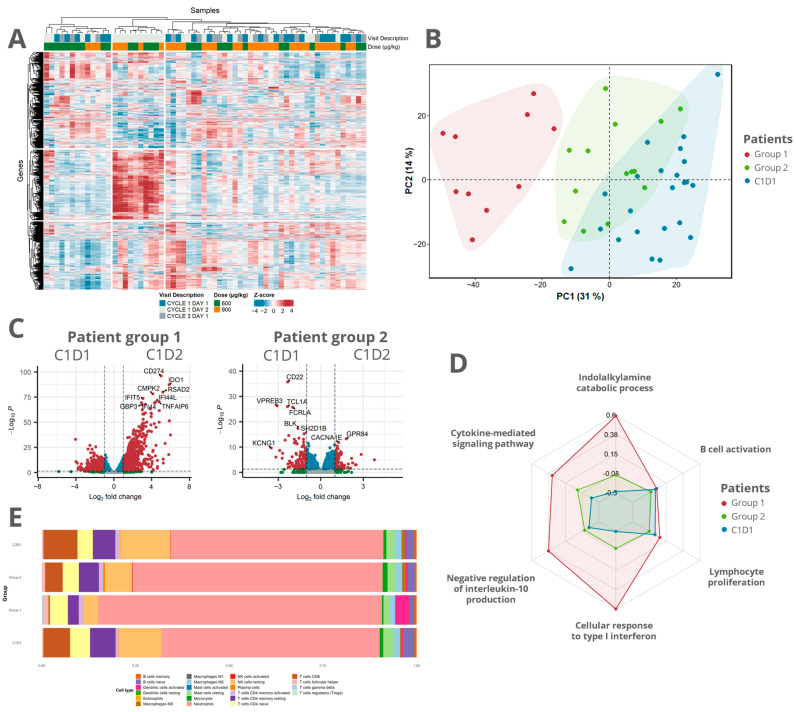
RNA sequencing data allowed for patient stratification with distinct mitazalimab-induced transcriptomic alterations in the patient groups: (**A**) Z-normalized VST transformed gene expression of the 1500 most variable genes across the 600 µg/kg and 900 µg/kg cohort. The top dendrogram shows the result of multiscale bootstrapping hierarchical clustering. One larger cluster (*p*-value < 0.05), containing samples at C1D2 treated with either 600 µg/kg or 900 µg/kg mitazalimab, was evident. This cluster was named patient group 1, and the rest of the samples were named patient group 2. (**B**) A similar clustering was seen in PCA of samples treated with 600 µg/kg or 900 µg/kg, at C1D1 and C1D2. (**C**) Volcano plots comparing C1D1 against C1D2 in patient groups 1 and 2. In general, a more prominent peripheral transcriptomic effect was seen in patient group 1. (**D**) Radar chart depicting the results of gene set variation analysis of selected GO pathways for the respective patient groups and samples at baseline. (**E**) Stacked bar plot of relative frequencies of the deconvoluted gene types from CIBERSORTx at C1D1 and C1D2 stratified based on patient group and C3D1.

Next, we investigated subject similarities across the dataset and applied hierarchical clustering using a multiscale bootstrap resampling of the 1500 most variable genes across all samples treated with 600 µg/kg or 900 µg/kg mitazalimab at C1D1, C1D2, and C3D1. By doing so, a significant cluster of 10 patients (named patient group 1) that had received mitazalimab at either 600 µg/kg or 900 µg/kg was apparent (*p* < 0.05). The remaining patients (*n* = 14, named patient group 2) did not cluster in a significant way (Figure 3A). The heatmap visualization of the gene expression of the 1500 most variable genes revealed a distinct gene cluster of upregulated genes in patient group 1 (Figure 3A). Pathway enrichment suggests that these genes were related to cytokine-mediated signaling in general and type I interferon (IFN) signaling in particular (Figure S4A). Dimension reduction based on the PCA of data points from C1D1 and C1D2 echoed the separation between the two patient groups at C1D2 along PC1 (31% variability; Figure 3B).

Mitazalimab was found to have a more pronounced effect on the peripheral transcriptome of patient group 1 than on that of patient group 2, with 1610 and 237 DEGs in the respective group (Figure 3C). The two patient groups shared 166 DEGs, which were mainly found to be significantly enriched in the cytokine-mediated signaling pathway (Figure S4B). Additionally, among the 1444 DEGs specific to patient group 1, the type I interferon signaling pathway was significantly enriched (Figure S4C). Gene set variation analysis (GSVA) echoed these findings, quantifying a higher expression of genes in the two aforementioned pathways as well as in genes related to the negative regulation of IL-10 (Figure 3D). The CIBERSORTx estimation of gene-type abundances showed a reduction in CD8^+^ T cells, resting NK cells, and B cells in both patient groups 1 and 2 at C1D2, albeit to a lesser extent in group 2. Furthermore, an increased abundance of activated dendritic cells was observed in patient group 1 (Figure 3E). Together, these findings indicate an immune activation in both patient groups but with the more noticeable transcriptomic alterations induced in patient group 1, mainly resulting from a type I interferon-like response.

**Figure 4:**
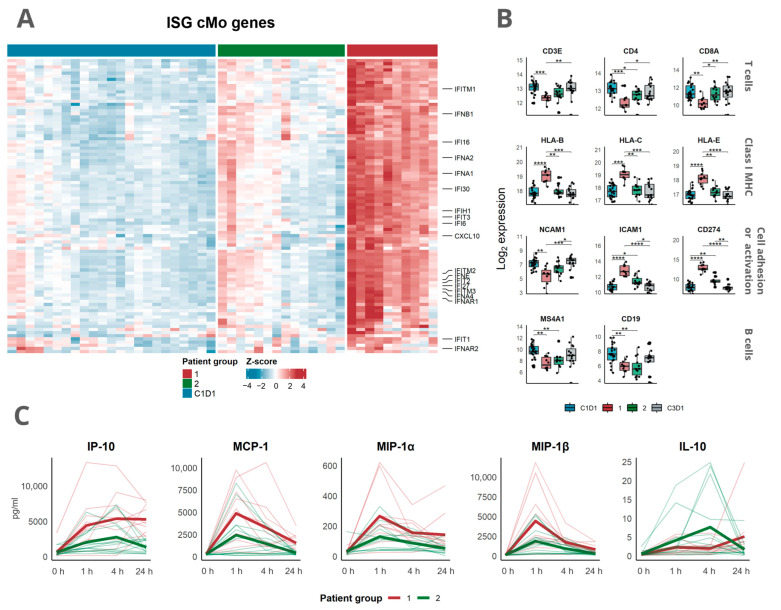
Mitazalimab-induced immune activation in patient groups stratified based on peripheral transcriptomic alterations: (**A**) Samples from patient group 1 showed higher expression of genes previously identified in interferon-stimulated monocytes (Table S4). The heatmap shows Z-score normalized VST gene expression with columns representing samples at either C1D1 or C1D2 stratified into patient groups. (**B**) Expression of immunologically relevant genes after mitazalimab treatment. Samples from C1D1 and C3D1 are grouped, and samples from C1D2 are stratified into identified patient groups. Dots show individual values. Dots represent individual values. * *p* < 0.05; ** *p* < 0.01; *** *p* < 0.001; **** *p* < 0.0001. (**C**) Serum cytokine levels after mitazalimab administration. Values are stratified based on patient groups identified from RNA sequencing data. Thick lines represent group means and thin lines indicate individual values.

To further evaluate the differences in immune responses between the groups, we first compared the expression of genes related to the regulation of interferon responses, by utilizing gene signatures previously identified in interferon-stimulated monocytes [29]. By doing so, a markedly higher expression of these genes was seen in patient group 1 compared with group 2 (Figure 4A). Second, the canonical immune-related transcripts were explored. An equal reduction in B cell-related transcripts (*CD19* and *MS4A1*) was seen in both patient groups (Figure 4B). Furthermore, a reduction in *CD3E* and *CD4* was observed in both groups, although mainly in group 1, whereas a reduction in *CD8A* was only seen in patient group 1. Moreover, an increase in transcripts related to MHC class I (*HLA-B*, *HLA-C*, and *HLA-E*) was only observed in patient group 1.

Next, we evaluated how the stratification based on transcriptomic profiles translated into blood cytokine levels. Following mitazalimab treatment, patients in group 1 seemed to have higher levels of IP-10, MCP-1, MIP-1α, and MIP-1β than those in group 2, who had higher levels of IL-10 (Figure 4C). This is in accordance with the transcriptomic data showing the overexpression of genes related to cell migration and the negative regulation of IL-10 production seen in patient group 1.

The analysis of baseline transcriptomic profiles in the two groups showed minor differences, mainly attributed to increased expression of ribosomal protein in group 2 (Figure S5). No significant differences in clinical characteristics, e.g., demographics and prior treatments, were identified between the groups (Table S3). However, patients in group 2 tended to have undergone more lines of treatment than those in group 1 (*p* = 0.054).

## 4. Discussion

Herein, we used RNA sequencing of peripheral blood from patients in a Phase I clinical trial with mitazalimab, a potent agonistic CD40 mAb, to describe early pharmacodynamic effects in patients with advanced solid tumors. Transient transcriptomic effects that reinforce the mode of action of mitazalimab, including the upregulation of genes related to type I interferon regulation and a relative reduction in genes in effector cell gene sets including CD8^+^ T cells, were seen at dose levels of 600 µg/kg and 900 µg/kg, supporting the dose selected for the ongoing Phase 2 study with mitazalimab (900 µg/kg, OPTIMIZE-1, NCT04888312). Overall, the pathways induced by mitazalimab support the potential of this antibody to overcome ICI resistance as well as overcome a suppressive tumor immune environment. Moreover, the transcriptomic data analysis allowed for patient stratification into groups where the gene expression induced by mitazalimab treatment was reflected in the cytokine response profiles.

Several clinical studies have reported the pharmacodynamic effects of agonistic CD40 mAbs determined via flow cytometry or cytokine analysis of peripheral blood. Typical flow cytometric findings include a transient reduction in CD19^+^ B cells with the activation of remaining CD19^+^ B cells determined through the upregulation of either CD80, CD86, or CD54 [14,15,16,17,30,31,32]. Increased serum cytokine release of MCP-1, IL-6, and tumor necrosis factor-alpha (TNFα) has been observed after intratumoral treatment [14]. In addition, elevation in cytokine levels in serum, including IFN-γ, MCP-1, MIP-1α, MIP-1β, IL-6, IL-8, IL-12, IP-10, and TNFα, has been detected after intravenous treatment [15]. However, to the best of our knowledge, no studies have yet analyzed the RNA sequencing data of peripheral blood for the pharmacodynamic effects of agonistic CD40 mAb. RNA sequencing provides an unbiased and global view of gene expression, allowing for a comprehensive assessment of the pharmacodynamic response [33]. The analysis presented herein demonstrates that the RNA sequencing-based analysis can both capture the known pharmacodynamic effects of mitazalimab [14,15] and detect novel findings. Biological systems are inherently complex, and the use of multiple complementary techniques is therefore advantageous to both validate findings and detect novel findings [34]. The data presented in this study support the finding that RNA sequencing could prove a valuable complement to flow cytometry and serum cytokine analysis in determining pharmacodynamic effects.

Binding to CD40, via CD40L, on APCs leads to the recruitment of TNF receptor-associated factor proteins, which in turn results in the release of nuclear factor-kappa B (NF-κB) transcription factors or the activation of mitogen-activated protein kinases. Ultimately, both pathways mediate various cellular responses, including the transcription of pro-inflammatory genes, such as various cytokines, that orchestrate the CD40-induced immune responses [35]. Indeed, we detected the strongest peripheral transcriptomic changes following mitazalimab treatment, which are related to cytokine production and regulation. Largely, these changes were attributed to type I interferon responses with induced expression of genes such as *OAS1*, *OAS2*, *IRF7*, and *MX1*. The dose–response relationship of mitazalimab reported in the present study is in line with assessments carried out on cytokine and exposure data [14,15,18]. Furthermore, the data suggest that pretreatment with corticosteroids immediately before mitazalimab treatment dampens the pharmacodynamic effects. These findings support the dosing regimen in the ongoing OPTIMIZE-1 study (NCT04888312), which does not include pretreatment with corticosteroids prior to the administration of mitazalimab.

Among the chemokine-related genes, *CCL2* (MCP-1), *CCL3L1* (MIP-1α), and *CCL8*, as well as *CXCL9*, *CXCL10* (IP-10), and *CXCL1*, were increased peripherally following mitazalimab treatment. Such changes suggest the activation of monocytes and dendritic cells after mitazalimab treatment. Many of these findings are in line with the reported increase in serum levels of, e.g., MCP-1, MIP-1α, and IP-10, after mitazalimab treatment [15], highlighting the relevance of the transcriptomic findings. Moreover, we observed a reduction in the peripheral expression of chemokine receptors (*CXCR3* and *CXCR5*), suggesting the extravasation of effector cells such as T cells and NK cells following mitazalimab treatment, which is also supported by our CIBERSORTx analysis and the results previously reported from flow cytometry analysis [15]. Furthermore, in line with the reported transient reduction in B cells and NK cells in the blood following agonistic CD40 treatment [15], we observed a reduction in both canonical B cell genes (e.g., *CD19*, *MS4A1*, *CD22*, *CD79A*, and *CD79B*) [36,37] and NK cell genes (e.g., *KIR3DL1*, *KIR2DL1*, and *KIR2DL2*) [38]. On the other hand, the increased expression of genes such as *CD54* and *CD274* suggests the activation of blood leukocytes [39,40], with increased expression of *LAMP3* and *CD177*, indicating the activation of DCs and neutrophils, respectively [41,42]. Taken together, these findings effectively support the proposed mode of action of mitazalimab.

Additionally, targeting CD40 in immunotherapy has been suggested to play a crucial role in remodeling the TME, especially via the repolarization of macrophages, leading to the degradation of tumor stroma by altering the expression of metalloproteases [10]. In this study, we detected an increase in *MMP1* and *MMP8*, as well as a marked increase in *ADAMTS2*, all of which are metalloprotease genes. Interestingly, we also observed a reduction in *MMP11* and *MMP28*. Possibly, this could indicate a shift in MMP expression rather than a global induction of MMPs, as previously postulated [10], which could potentially mediate the degradation of fibrosis associated with certain malignancies making them more permeable for both immune cells and medications.

The pharmacodynamic effects of mitazalimab have been preclinically evaluated in tumor-bearing human CD40-transgenic mice [13,18]. Notably, peripheral transcriptomic changes upon mitazalimab treatment in this preclinical in vivo model correlate well with the clinical transcriptomic changes. Thus, this finding strengthens the translation of the preclinical findings of mitazalimab in the preclinical model.

The large volume of data obtained through RNA sequencing allowed us to stratify patients based on their pharmacodynamic response, which was not possible from flow cytometric or cytokine analysis [15]. Notably, the changes seen on a transcriptomic level in the patient groups also correlated with cytokine levels in the respective groups. A non-significant trend indicates that patients with more previous lines of therapy seemed to have a lower peripheral immune activation. Speculatively, this could be due to systemic immune alteration from, e.g., chemotherapy or ICI. However, the small sample size and diverse patient population (*n* = 23) limit the interpretation, and additional studies would be warranted to investigate if these findings remain valid in specific indications and first-line treatment settings. In addition, a correlation between peripheral transcriptomic changes to both intratumoral immune changes and clinical response is yet to be determined.

In summary, by using RNA sequencing on peripheral blood from patients in clinical trials with mitazalimab, we could confirm the pharmacodynamic effects of mitazalimab previously observed with flow cytometry and serum cytokine analysis. Furthermore, the larger volume of data obtained using RNA sequencing, as compared to previous flow cytometry and serum cytokine data, revealed a distinct response pattern in a subset of patients that was echoed in the serum cytokine data. In conclusion, the data presented herein reinforce the mode of action of mitazalimab and support its further investigation in clinical trials of solid malignancies.

## Figures and Tables

**Table 1:** Demographics and baseline clinical characteristics of all included patients.

Parameter		Number of Patients
*n*		38 *
Age, years (median (range))		56 (18–77)
Sex, *n* (%)	Female	19 (50.0)
	Male	19 (50.0)
Corticosteroid pretreatment, *n* (%)	No	33 (86.8)
	Yes	5 (13.2)
Dose (µg/kg), *n* (%)	75	3 (7.9)
	200	5 (13.2)
	600	16 (42.1)
	900	14 (36.8)
Cancer type, *n* (%)	Adrenal	1 (2.6)
	Breast	4 (10.5)
	Cervical	2 (5.3)
	Cholangiocarcinoma	3 (7.9)
	Choroidal melanoma	1 (2.6)
	Colorectal	3 (7.9)
	Melanoma	2 (5.3)
	Neuroendocrine	1 (2.6)
	NSCLC	3 (7.9)
	Pancreas	3 (7.9)
	Rectal cancer	1 (2.6)
	Renal cancer	1 (2.6)
	Salivary gland	3 (7.9)
	Sarcoma	5 (13.2)
	Testicular	1 (2.6)
	Thymus	3 (7.9)
	Thyroid	1 (2.6)
Prior lines of treatment, *n* (%)	0–4	30 (78.9)
	>4	8 (21.1)
	Median (range)	3 (0–9)

* Subset of patients included in Phase I trial NCT02829099; NSCLC: non-small cell lung cancer.

## Data Availability

The data presented in this study, together with R scripts that replicate the analysis outlined herein, will be available in a repository on GitHub upon request from the corresponding author.

## Supplementary Materials

The following supporting information can be downloaded at: https://www.mdpi.com/article/10.3390/cells12192365/s1, Figure S1: DEGs between C1D1 and C1D2 at 75 and 200 µg/kg mitazalimab; Figure S2: Correlation between transcriptomic changes in human and mice following mitazalimab treatment; Figure S3: Effect of corticosteroid pretreatment; Figure S4: Pathway enrichment in patient groups; Figure S5: Baseline transcriptomic differences between patient group 1 and 2; Table S1: DEGs and GO after mitazalimab treatment; Table S2: DEGs between mitazalimab with or without CS pretreatment; Table S3: Patient characteristics based on patient group; Table S4: ISG cMo gene list.
