# Supplementary material for: Early Pharmacodynamic Changes Measured Using RNA Sequencing of Peripheral Blood from Patients in a Phase I Study with Mitazalimab, a Potent CD40 Agonistic Monoclonal Antibody
Source: Cells. 2023 Sep 27;12(19):2365. doi: 10.3390/cells12192365 (PMC10572020; doi:10.3390/cells12192365)
Supplement: Supplementary file 1 [file cells-12-02365-s001.zip › Supplementary figures.pdf]

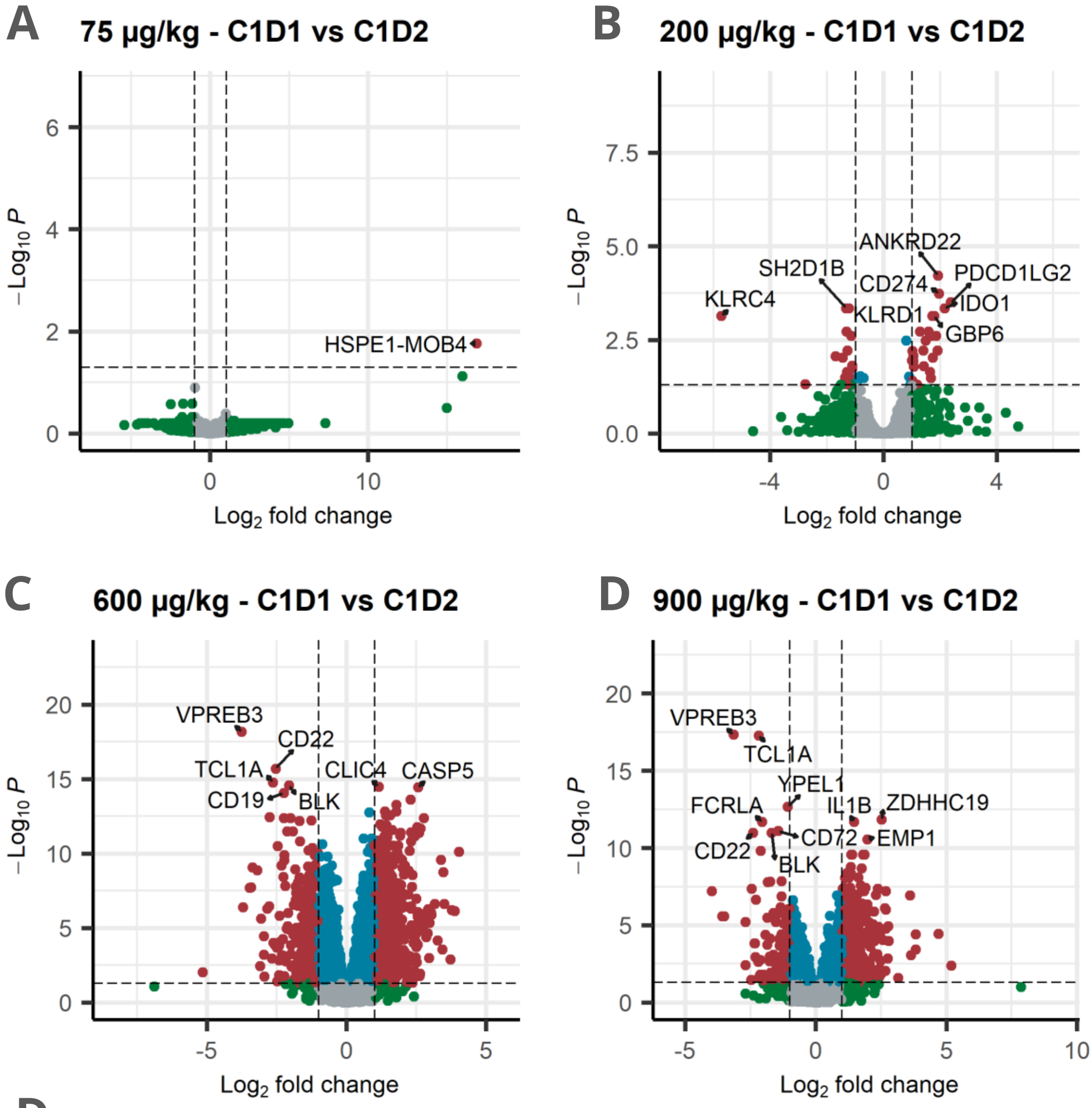


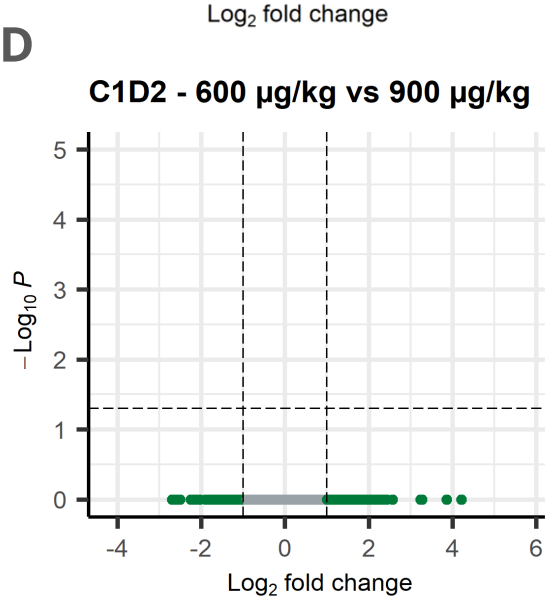


**Figure S1.** A-E) DEG between C1D1 and C1D2 at all dose levels of mitazalimab. D) DEG at C1D2 between patients treated with 600 µg/kg or 900 µg/kg mitazalimab.

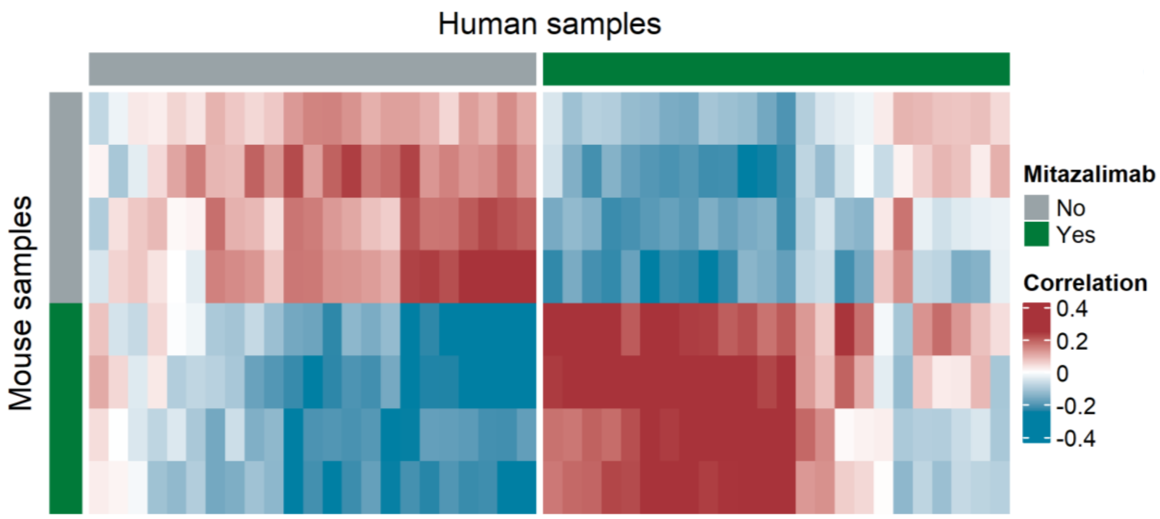


**Figure S2.** Correlation between transcriptomic changes in human and mice following mitazalimab treatment. Patient dosed with 600 µg/kg or 900 µg/kg mitazalimab was included as human samples. Mice were hCD40-transgenic inoculated subcutaneously with MB49 cells. The mice were treated with mitazalimab or vehicle 10-11 days after inoculation and samples blood samples were collected 24 hours post-treatment, RNA was prepared and sequenced. Mice samples underwent the same analysis pipeline as human samples. DEG in human was calculated between C1D1 and C1D2 while DEG in mice were calculated between mice treated with mitazalimab and untreated mice. DEG in human and mice was combined and the correlation between samples were based on z-score of VST normalized expression and was calculated using Pearson correlation.

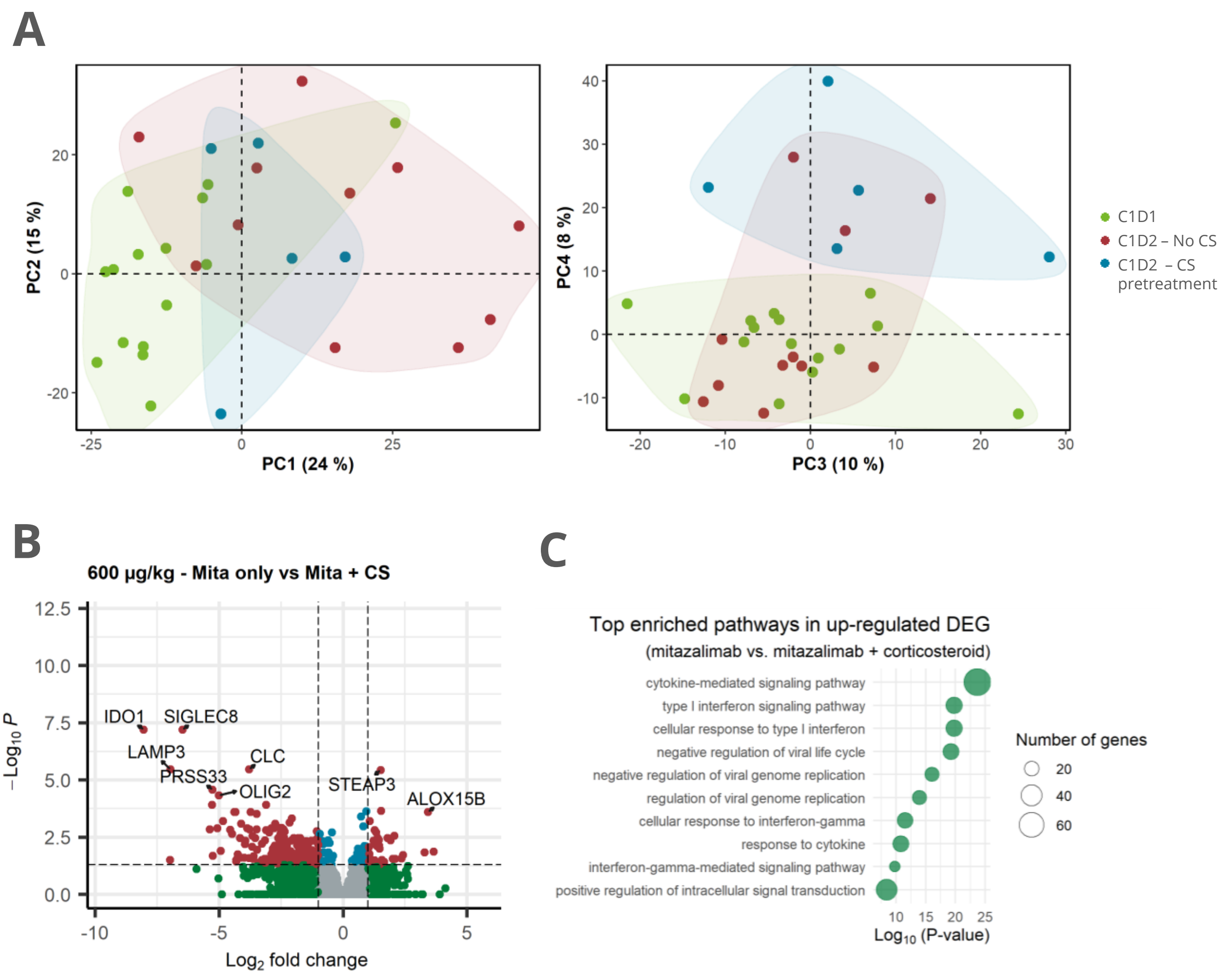


**Figure S3.** Effect of corticosteroid pretreatment. A) Shows PCA plots of samples treated with 600 µg/kg mitazalimab with or without CS pretreatment, at C1D1 and C1D2. The mitazalimab-induced separation along PC1 is reduced with CS pretreatment, and a separation of CS pretreated samples are seen along PC4. B). Volcano plot of DEG at C1D2 between samples pretreated with or without CS. Samples pretreated with CS had a reduced transcriptomic inflammatory response to mitazalimab. C) Enriched GO terms based on DEG in B). DEG in samples not pretreated with CS were mainly related to cytokine-mediated signaling and response to type I interferon.

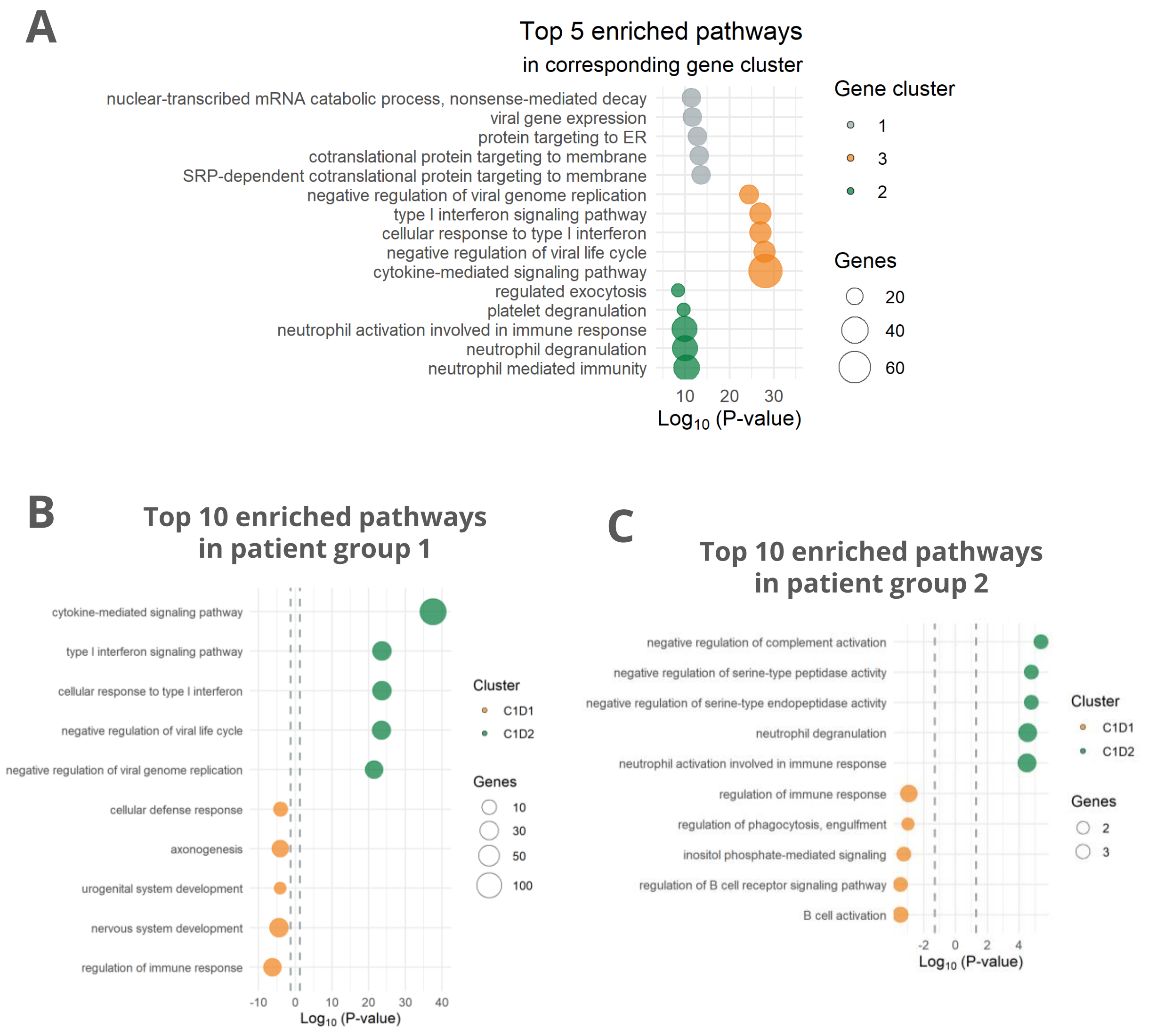


**Figure S4.** Pathway enrichment in patient groups. A) Shows enriched GO terms from genes in respective gene cluster in heatmap from Figure 3A. B) Shows enriched GO terms in DEG in patient group 1 between C1D1 and C1D2. C). enriched GO terms from genes in respective gene cluster in heatmap from Figure 3A. B) Shows enriched GO terms in DEG in patient group 2 between C1D1 and C1D2.

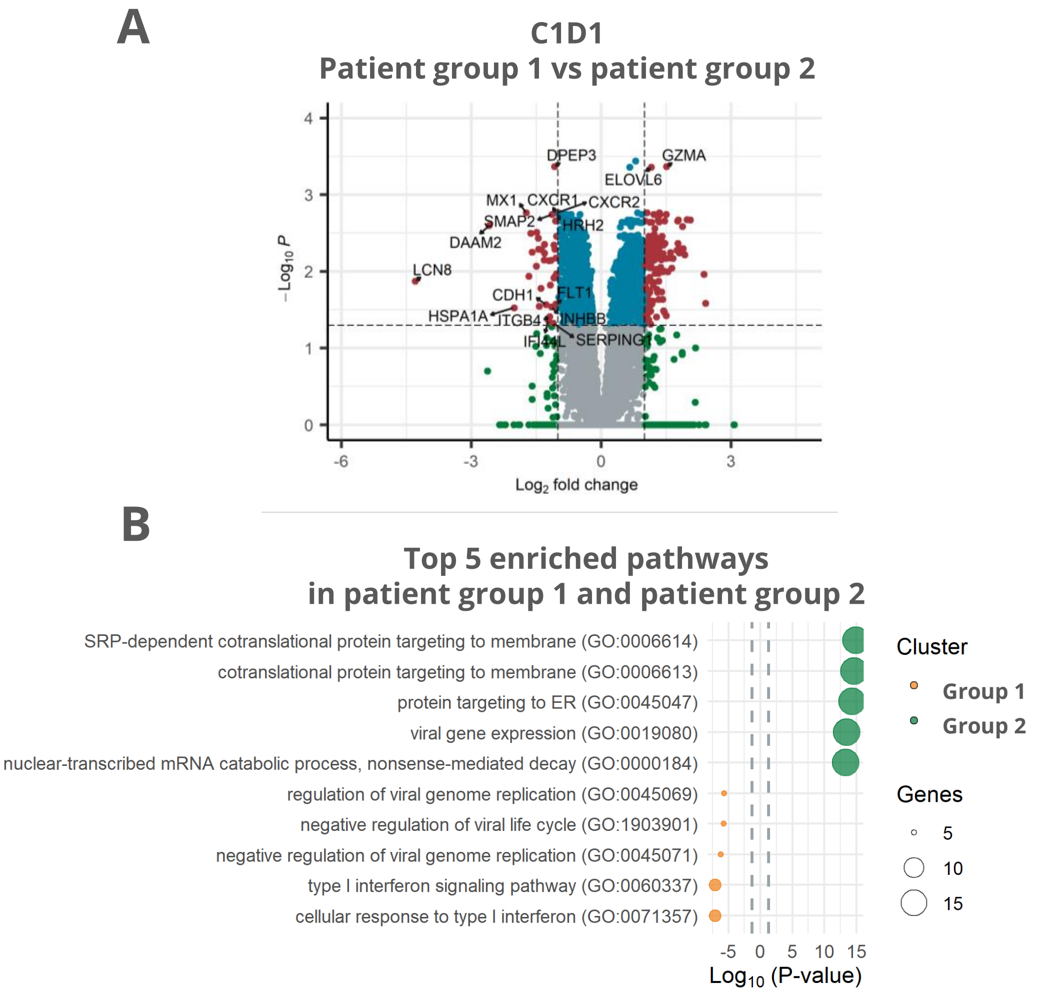


**Figure S5**. Baseline transcriptomic differences between patient group 1 and 2. A) Shows volcano plot of DEG between patient group 1 and 2 at C1D1. B) Shows enriched GO terms of up-regulated DEG in respective patient group at C1D1. Patient group 2 showed higher expression of ribosomal genes, as compared to patient group 1.
