# Supplementary material for: Early Pharmacodynamic Changes Measured Using RNA Sequencing of Peripheral Blood from Patients in a Phase I Study with Mitazalimab, a Potent CD40 Agonistic Monoclonal Antibody
Source: Cells. 2023 Sep 27;12(19):2365. doi: 10.3390/cells12192365 (PMC10572020; doi:10.3390/cells12192365)
Supplement: Supplementary file 1 [file cells-12-02365-s001.zip › Table S3. Patient characterisitcs based on patient group.pdf]

Table S3. Patient characteristics stratified on patient group.

		**Patient group 1**	**Patient group 2**	*p*
n		9	13	
Dose (μg/kg), n (%)	600	5 (55.6)	6 (46.2)	1.000
	900	4 (44.4)	7 (53.8)	
Cancer type, n (%)	Adrenal	0 (0.0)	1 (7.7)	0.418
	Breast	1 (11.1)	2 (15.4)	
	Cervical	1 (11.1)	1 (7.7)	
	Cholangiocarcinoma	1 (11.1)	2 (15.4)	
	Colorectal	0 (0.0)	3 (23.1)	
	Melanoma	1 (11.1)	0 (0.0)	
	NSCLC	0 (0.0)	1 (7.7)	
	Pancrease	2 (22.2)	0 (0.0)	
	Rectal	0 (0.0)	1 (7.7)	
	Renal	1 (11.1)	0 (0.0)	
	Salivary gland	0 (0.0)	1 (7.7)	
	Sarcoma	1 (11.1)	1 (7.7)	
	Testicular	1 (11.1)	0 (0.0)	
Age (median (IQR))		62 (55, 66)	53 (49, 56)	0.115
Sex, n (%)	Female	5 (55.6)	7 (53.8)	1.000
	Male	4 (44.4)	6 (46.2)	
Prior lines of treatment, n (%)	0-4	9 (100)	8 (61.5)	0.054
	> 4	0 (0)	5 (38.5)	
	Median (range)	3 (2-4)	3 (0-9)	

* NSCLC: non-small cell lung cancer
